# Neonatal Morbidities of Fetal Growth Restriction: Pathophysiology and Impact

**DOI:** 10.3389/fendo.2019.00055

**Published:** 2019-02-07

**Authors:** Atul Malhotra, Beth J. Allison, Margie Castillo-Melendez, Graham Jenkin, Graeme R. Polglase, Suzanne L. Miller

**Affiliations:** ^1^Monash Newborn, Monash Children's Hospital, Melbourne, VIC, Australia; ^2^The Ritchie Centre, Hudson Institute of Medical Research, Melbourne, VIC, Australia; ^3^Department of Paediatrics, Monash University, Melbourne, VIC, Australia; ^4^Department of Obstetrics and Gynaecology, Monash University, Melbourne, VIC, Australia

**Keywords:** IUGR, FGR, bronchopulmonary dysplasia, cardiac, brain injury, necrotizing enterocolitis

## Abstract

Being born small lays the foundation for short-term and long-term implications for life. Intrauterine or fetal growth restriction describes the pregnancy complication of pathological reduced fetal growth, leading to significant perinatal mortality and morbidity, and subsequent long-term deficits. Placental insufficiency is the principal cause of FGR, which in turn underlies a chronic undersupply of oxygen and nutrients to the fetus. The neonatal morbidities associated with FGR depend on the timing of onset of placental dysfunction and growth restriction, its severity, and the gestation at birth of the infant. In this review, we explore the pathophysiological mechanisms involved in the development of major neonatal morbidities in FGR, and their impact on the health of the infant. Fetal cardiovascular adaptation and altered organ development during gestation are principal contributors to postnatal consequences of FGR. Clinical presentation, diagnostic tools and management strategies of neonatal morbidities are presented. We also present information on the current status of targeted therapies. A better understanding of neonatal morbidities associated with FGR will enable early neonatal detection, monitoring and management of potential adverse outcomes in the newborn period and beyond.

## Overview and description

Fetal growth restriction (FGR) describes the fetus that does not grow to its expected biological potential *in utero*, and is a relatively common complication of pregnancy. True FGR, as compared to constitutional smallness, is a pathological condition wherein the placental fails to deliver an adequate supply of oxygen and nutrients to the developing fetus, termed placental insufficiency. As a consequence, fetal growth becomes stunted. It is only in the last several years that consensus definitions for pathological FGR have been developed (1), but it remains that many cases of FGR *in utero* remain undetected, and therefore the neonatal description of small for gestational age (SGA) continues to be a useful and necessary proxy for FGR (2). Traditionally, an estimated fetal weight or abdominal circumference of less than the 10th centile for the population at a given gestational age was considered highly suggestive of FGR. However this broad description of SGA includes the many infants (~20%) that are born small, but are otherwise healthy (2). Accordingly, consensus definitions for FGR now incorporate Doppler indices of placental function/ dysfunction during pregnancy (1), to provide a more robust assessment of pathological fetal growth restriction. Clear and well-defined guidelines for description of FGR subsequent to placental insufficiency are important for two broad reasons, (i) early identification of FGR flags infants who are at significantly elevated risk for neonatal complications, and (ii) early identification of infants with FGR who would benefit from intervention(s) to improve outcomes. The etiology of many adverse consequences of FGR arise *in utero* from fetal hypoxia and nutrient deprivation secondary to placental dysfunction, with fetal hemodynamic adaptations *in utero* laying the foundation for altered organ structure and function in the neonatal period and beyond.

## Etiology and Uteroplacental Factors

The basic determinants of fetal growth are the individual's genetic makeup, nutrient availability from the mother, and environmental factors, coupled with the capacity of the placenta to adequately transfer nutrients and oxygen to the fetus, and endocrine modulation of these interactions (3, 4). Reduced fetal growth, and subsequent pathological FGR, can be caused by maternal factors (e.g., under nutrition, hypertension, preeclampsia), fetal (chromosomal abnormalities, multiple fetuses) or placental factors (5), however in the majority of cases, FGR results from placental dysfunction (6). Here, the term *placental insufficiency* is broadly used to describe reduced transfer of oxygen and nutrients to the fetus, with adverse effects on fetal development. Antecedents of placental insufficiency can include maternal malnutrition and hypertension, but in up to 60% of cases the placental insufficiency is idiopathic, wherein there is a physiological deficiency in the remodeling of uterine and placental spiral arteries resulting in restricted uteroplacental perfusion (7).

Abnormalities in placental function provide a primary clinical indicator that transfer of oxygen and nutrients is suboptimal, and fetal growth may be adversely affected. In the fetus, placental insufficiency is characterized by preferential blood flow redistribution to the vital organs (brain, myocardium, and adrenal glands), while other organs, including the gastrointestinal tract, skin, and others may be deprived of sufficient blood flow. This fetal redistribution of blood flow occurs as a direct result of hypoxia, and can be detected as altered umbilical, uterine and/or middle cerebral artery Doppler flows (8). Large population studies of small but otherwise healthy infants at birth (Apgar ≥ 7 at 5 min of life) demonstrates that severely growth restricted infants at the third birth weight centile are indeed chronically hypoxic; umbilical vein median pO_2_ 13 mmHg (FGR) versus 26 mmHg (normally grown infants), and median SaO_2_ 16 vs. 55% respectively (9, 10).

In addition to the fundamental roles of oxygen and glucose for development, fetal growth is dependent on a number of key anabolic hormones—placental, pancreatic, thyroid, adrenal and pituitary hormones—any disruption in these can also lead to FGR (11, 12). The insulin-like growth factors -I and -II (IGF-I and IGF-II) are both proposed to play central roles in normal fetal growth, stimulating fetal cell proliferation, differentiation, protein and glycogen synthesis, where these actions are mediated via their receptors and the IGF-binding proteins (IGFBPs). The two IGFs are detected in the fetal circulation in early gestation, and in particular it is noted that decreased serum IGF-1 is correlated with reduced fetal growth (3, 13). IGF-1 also has a central role in brain growth, white matter development and brain connectivity (14). Pregnancy-associated plasma protein-A (PAPP-A), secreted by the placental decidua, cleaves IGFBP-4, which in turn is a potent inhibitor of IGF bioactivity. Accordingly, low levels of PAPP-A in early pregnancy are linked with an increased risk for FGR, although the predictive value of this biomarker still remains poor (15). A recent study has investigated whether administration of IGF-1 into the amniotic fluid can improve postnatal growth and metabolism in a sheep model of FGR, and results from this study look promising (16) (see Interventions for Improved Outcomes section). Glucocorticoid hormones play a central role in the development and maturation of fetal organs, while growth hormone, which is the major hormonal regulator of postnatal growth, has no demonstrable effect on fetal growth *per se* (17). Exogenous glucocorticoids are administered to pregnant women at imminent risk of preterm birth to mature the fetal lungs, and preterm birth is a common complication of FGR. Preclinical and clinical evidence demonstrates that antenatal steroids may exacerbate growth restriction (particularly repeat doses) (18) and that the FGR fetus differentially responds to antenatal steroids compared to appropriately-grown fetuses, likely mediated via altered placental response to steroids (19). Antenatal glucocorticoids may not significantly improve neonatal outcomes in FGR preterm infants (20), and indeed, may have adverse effects on brain development (21, 22). Further research is clearly needed in this area.

The fetus mounts a critical hemodynamic response to hypoxia, aimed at ensuring the most important fetal organs maximize their oxygen supply. This adaptive response redistributes blood flow away from peripheral vascular beds which is preferentially shunted toward essential organs, termed *brain sparing* (23). This results in preferential supply of blood flow to favor the brain, heart, and adrenals, at the expense of the gut, kidney, hematologic organs, and peripheral vascular beds. When fetal hypoxia is chronic in nature, as occurs with placental insufficiency, the persistent fetal hemodynamic shift has significant consequences for the fetus and neonate. Characteristically, prolonged fetal hypoxia reduces fetal weight overall, but also does so in an asymmetric manner, with relatively spared head size and a thin and/or shorter body length. While hemodynamic redistribution may be an attempt to protect vital organs from hypoxic injury, an adverse impact on fetal organ development and vascular remodeling is increasingly being recognized (23, 24). For example, the shunting of blood flow away from the kidneys is now recognized as contributing to suboptimal renal development with reduced nephron endowment (25). Further, sustained vasoconstriction of peripheral vascular beds alters local arterial wall properties including endothelial vasodilator dysfunction and sympathetic hyperinnervation, and consequently contributes to cardiac remodeling (26). The short and long-term consequences of sustained redistribution of cardiac output are profound, for both spared and non-spared organs, and these will be discussed in more detail below.

The overall incidence of FGR depends on the diagnostic criteria used, and the population being examined. It is estimated that between 3 and 9% of pregnancies in the developed world, and up to 25% of pregnancies in low-middle income countries are affected by FGR (27, 28). Factors that influence FGR rates in communities include maternal nutrition, maternal and paternal smoking rates, alcohol and drug addiction, socio-economic status, maternal activity, stress during pregnancy and genetic make-up (29). The incidence of FGR is significantly higher in low- and middle- income countries, compared to high-income countries, and this is notably contributed by a large number of FGR infants born in the Asian continent, which accounts for approximately 75% of all affected infants in the world, followed by Africa and South America (30).

## Classification Types of FGR

FGR can be classified as early- or late-onset, reflecting the gestational age when growth restriction is diagnosed. Early onset FGR (<32 weeks gestation) is the more severe phenotype, associated with significant disruption to placental perfusion leading to chronic fetal hypoxia, and with subsequent fetal cardiovascular adaptation *in utero* (31). Fetuses with early-onset placental insufficiency are more likely to be born preterm, to deteriorate over weeks, and have a high risk of morbidity or mortality. Late onset FGR (≥32 weeks gestation) is the more common presentation of growth restriction (up to 80% of FGR cases), and is generally linked with a milder placental deficit, together with a lesser degree of fetal hemodynamic adaptation. Although placental dysfunction is mild, this group has a high risk of deteriorating rapidly, such that they have an elevated risk of stillbirth (31). This broad distinction between early- and late-onset FGR demonstrates that the timing when placental function becomes rate limiting for the fetus is a principal factor affecting outcome.

**Figure 1:**
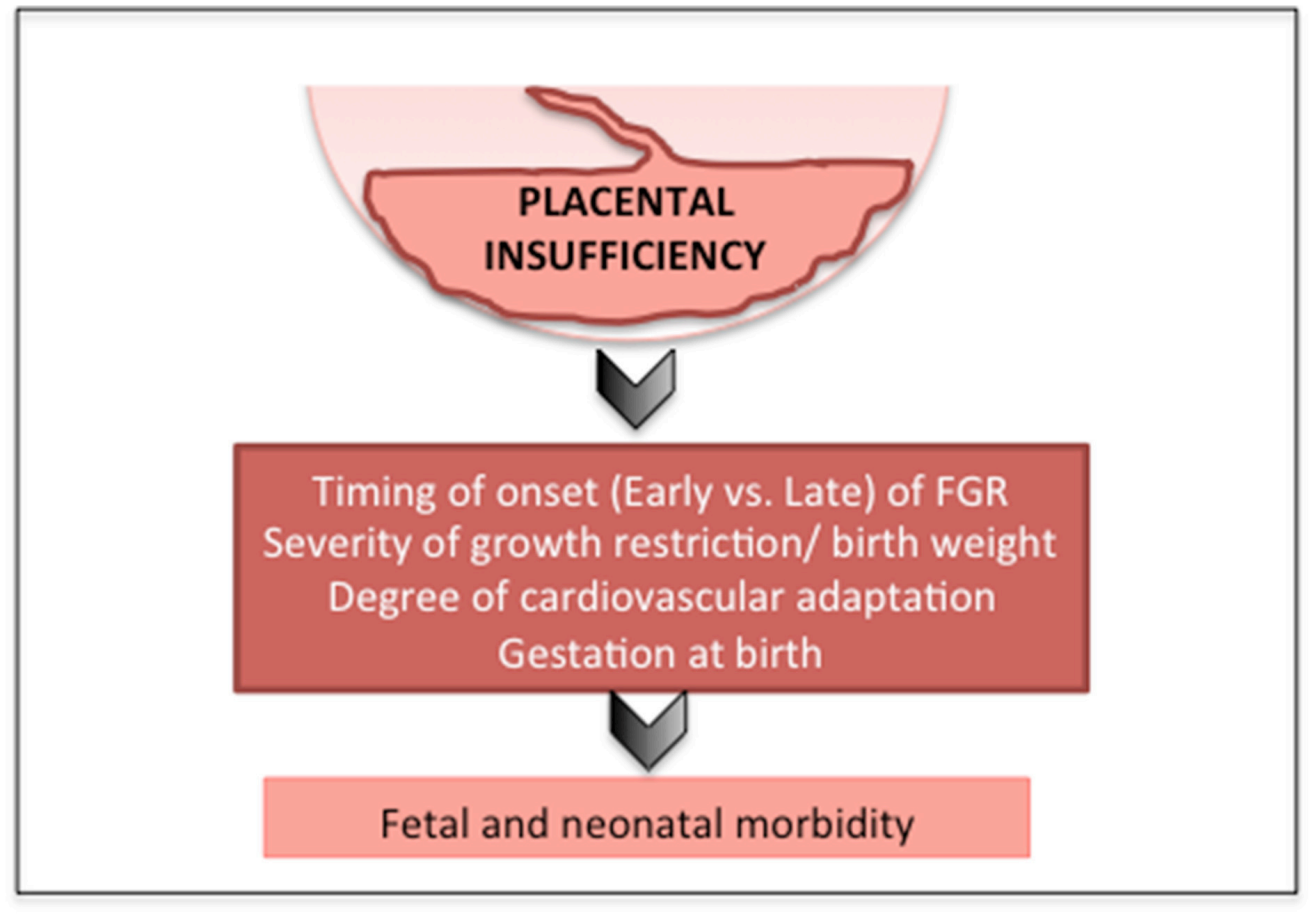
Factors that affect outcomes in FGR.

Advances in obstetric monitoring mean that it is increasingly likely that placental insufficiency and fetal growth restriction are detected during pregnancy. However, a significant proportion (up to 50%) of FGR fetuses remain undiagnosed, and are first recognized only very late in pregnancy or at birth (32–34). Furthermore, debate continues around the utility of third trimester ultrasound for the detection of late-onset FGR (35), with a recent study reporting that undiagnosed FGR does not lead to increased incidence of morbidity in neonates (36). These data likely reflect that it is predominantly the early-onset FGR infants with severe placental insufficiency, and worse neonatal outcomes, who are more straightforward to detect during pregnancy. Currently, no effective antenatal therapy exists for FGR, hence, delivery of the fetus remains the only viable option for a severely affected pregnancy; this often occurs preterm, introducing further risk of morbidity and mortality (37, 38). Together these data are indicative that the timing of the onset of placental insufficiency (early vs. late), gestation at birth, and severity of compromise/birth weight are the most predictive factors for neonatal outcomes (39) (Figure 1).

## Perinatal Morbidities

**Figure 2:**
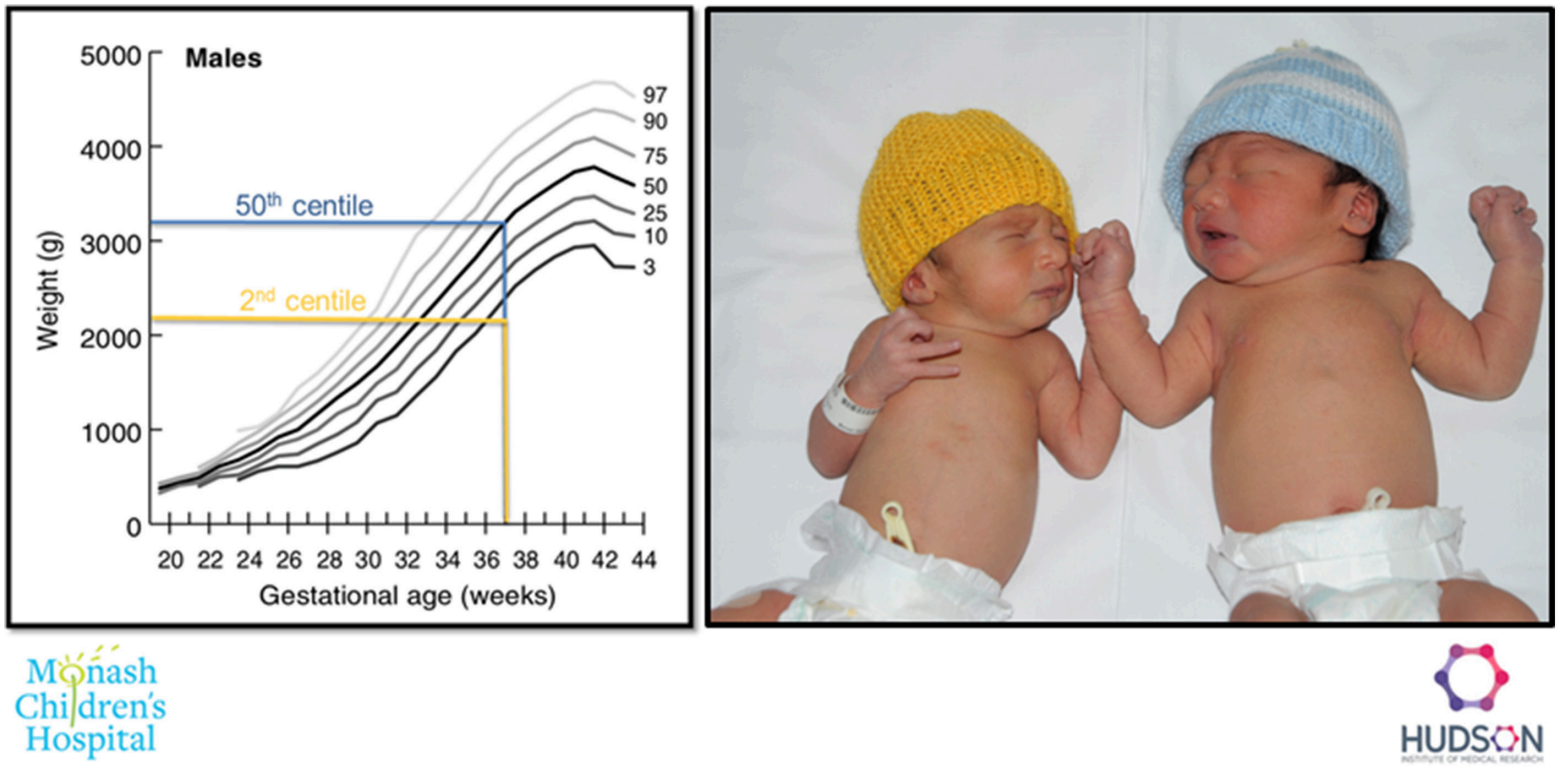
Example of a FGR (2nd centile weight for age, yellow), and an appropriately grown (50th centile weight for age, blue) infant born at 37 weeks gestation.

**Figure 3:**
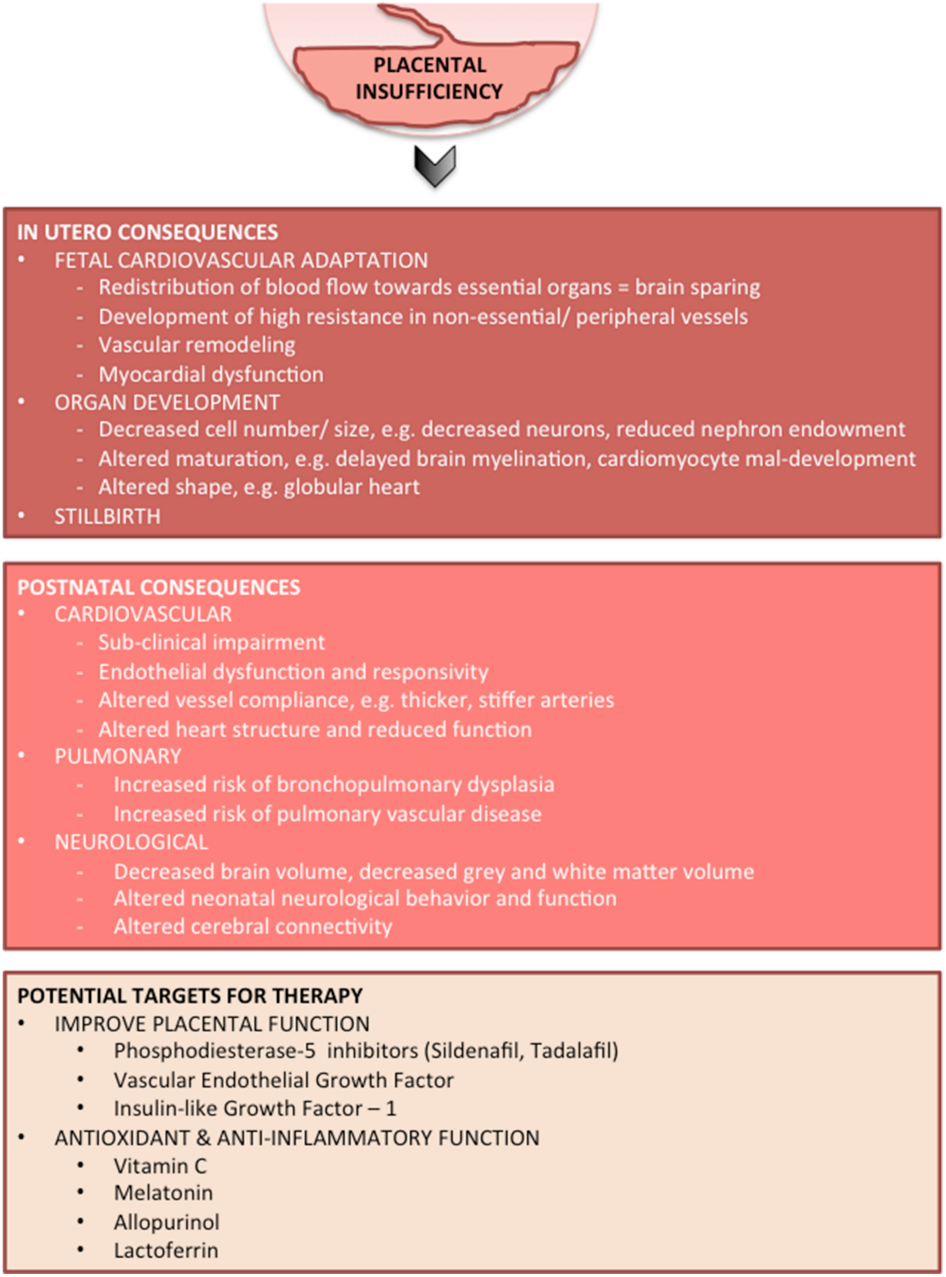
*In-utero* and postnatal consequences of FGR and potential targeted therapies.

A typical FGR infant at term age and an appropriately grown infant at term are shown in Figure 2. Key pathophysiological mechanisms driving fetal growth restriction and the resulting *in-utero* and postnatal consequences are highlighted in Figure 3. Placental pathology and FGR are strongly associated with fetal demise *in utero*, and stillbirth (40–42). FGR is the greatest risk factor for stillbirth; overall it is shown that up to 50% of infants who are stillborn were small for gestational age or growth restricted (43). The detection, early diagnosis, surveillance and delivery of the severely growth restricted fetus are paramount to decrease stillbirth, but it remains that 40% of severe FGR infants (<3rd centile for birth weight) remain undetected *in utero* (44).

After birth, FGR infants are more likely to spend a significantly longer time in NICU compared to gestation age-matched infants (45). Accordingly, financial costs associated with the care of FGR infants are high, given that many of them will remain in NICU for prolonged periods (46, 47). FGR infants demonstrate elevated rates of intolerance to feeds/ milk, feeding difficulties and necrotizing enterocolitis (NEC). NEC is predominantly seen in infants who are born preterm, but late preterm infants are more likely to develop NEC if they were growth restricted (48). It is likely that i*n utero* chronic fetal hypoxia and subsequent cardiovascular redistribution of blood flow away from the gastrointestinal tract contribute to immature gut development (49). FGR newborns, especially with abnormal flows in the umbilical artery prior to birth, are shown to have more feed intolerance when compared to their well-grown preterm counterparts (50). Superior mesenteric artery blood flows have been used as a marker for splanchnic perfusion in neonates and decreased flows correlate with feed intolerance (51). Application of near infra-red spectroscopy in the neonatal period as an assessment tool for monitoring gut perfusion can detect changes in splanchnic oxygen delivery, which may be reduced in FGR infants and may predict feeding intolerance and development of NEC (52). Studies have shown that preterm FGR infants do not tolerate enteral feeds in the first few days of life (53) but conversely there is evidence that delaying enteral feeds in preterm FGR infants does not confer any protection against feed intolerance or NEC (54). In fact, it may delay establishment of feeds and increase length of stay in the neonatal unit (55).

Malnutrition and low birth weight puts FGR infants at an increased risk of a number of transient neonatal morbidities including hypothermia, altered glucose metabolism (hypoglycemia, hyperglycemia), hypocalcemia, polycythemia, jaundice and sepsis (5). Increased risk of infection is also common, potentially related to depressed immunological state and competence (56). FGR infants born preterm also have an increased risk of retinopathy of prematurity (57). FGR is linked to altered nephrogenesis, due to suboptimal tubular development caused by intrauterine hypoxia (58), and in turn, urinary Cystatin-C excretion is increased in FGR infants compared to appropriately-grown infants which is seen to reflect reduced renal volume (59). It is therefore suggested that increased secretion of Cystatin-C signifies nephron loss as a result of the negative impact of FGR on kidney development. Factors involved in nephron loss may include intrauterine hypoxia, decreased antioxidant capacity, and altered levels of growth factors.

## Specific Neonatal Morbidities (Table 1)

### Cardiovascular Morbidity

#### Clinical Features

In addition to chronic hypoxia, placental insufficiency imposes other important stressors for the developing fetus, such as oxidative stress, inflammation and increased hemodynamic stress. This leads to elevated cardiac afterload due to high placental vascular resistance, which in turn directly and indirectly impacts on the developing cardiovascular system. It is now accepted that the fetal adaptations to these combined stressors sets the fetus, and future offspring, on a path of predetermined increased risk of cardiovascular disease (60, 61). It is also now apparent that subclinical or subtle evidence of cardiovascular dysfunction is present in fetal and/or early neonatal life, well before the onset of significant cardiovascular or metabolic disease in adulthood, supporting the notion of perinatal programming (60).

**Table 1:** Neonatal morbidities in fetal growth restriction.

	**Cardiovascular morbidity**	**Respiratory morbidity**	**Neurological morbidity**	**Others**
Neonatal period	Early hypotension Persistent fetal circulation/PPHN Structural heart changes Vessel wall rigidity Cardiac function issues Late systemic hypertension Secondary pulmonary hypertension	Increased need for respiratory/ventilator support Meconium aspiration syndrome Pulmonary hemorrhage Bronchopulmonary dysplasia	Perinatal asphyxia Microcephaly Cranial ultrasound abnormalities (IVH, PVL) White matter and gray matter changes on MRI Functional and DTI MRI changes General movement assessment abnormalities EEG abnormalities	Poor transition Hypoglycemia Hypocalcemia Hypothermia Sepsis Jaundice Polycythemia Prolonged NICU stay Feed intolerance Delay in establishment of feeds Necrotizing enterocolitis Renal tubular injury Retinopathy of prematurity
Long term impact	Hypertension Ischemic heart disease Stroke Atherosclerosis	Chronic respiratory insufficiency Reactive airway disease	Neurodevelopmental issues Behavioral problems Learning difficulties Cerebral palsy Dementia Mental health issues	Failure to thrive Obesity Immune dysfunction Osteoporosis Metabolic syndrome Renal issues Hormonal issues Cancer Shortened life span

*PPHN, persistent pulmonary hypertension; IVH, intraventricular hemorrhage; PVL, periventricular leukomalacia; MRI, magnetic resonance imaging; DTI, diffusion tensor imaging; EEG, electroencephalography; NICU, neonatal intensive care unit*.

Advances in Doppler ultrasonography of the placental and fetal circulations provide a window of opportunity to observe and quantify fetal cardiovascular function, and early dysfunction. In early-onset FGR, severe placental insufficiency is characterized by high vascular resistance within placental vascular beds, resulting in absent or reversed diastolic umbilical artery flow, as well as high pulsatility index in the ductus venosus and increased dilation of cerebral vessels evident of fetal brain sparing (31). In late-onset FGR, umbilical artery flow may be normal, representing a milder placental insufficiency. Despite this, brain sparing is still evident, with increased cerebral to placental blood flow driven primarily by vasodilation within the middle cerebral artery in response to hypoxia (62). In both early- and late-onset FGR, increasing vasodilation within cerebral vascular beds is indicative of a worsening fetal state (63). Increased myocardial performance index, an index incorporating both diastolic and systolic function to assess global cardiac function/dysfunction, is evident from 24 weeks gestation in early onset-FGR fetuses (64). An increase in myocardial performance index is not indicative of improved performance, but rather demonstrates an increased time of systolic relaxation evident in early-onset FGR. Increased myocardial dysfunction is also present in late-onset FGR from >35 weeks gestation. In this population, late-onset placental insufficiency and FGR results in fetuses with larger, more globular hearts and early indices of functional deficits with impaired relaxation (65). This study is the first to show that late-onset placental insufficiency and FGR induces cardiac dysfunction that is detectable in the third trimester of pregnancy (~35 weeks gestation), indicating the presence of cardiac programming prior to birth. It has also been shown that cardiac dysfunction and markers of cardiac injury such as BNP and H-FABP become increasingly worse as the severity of fetal compromise progresses (66).

#### Pathophysiology

In the presence of very high placental resistance associated with a sub-optimal pregnancy, the fetal heart contracts against an increased afterload, thereby increasing the work required to contract with each beat, resulting in increased heart wall stress and hypertrophy (65). Over a sustained period, hypertrophy increases wall thickness altering ventricular compliance. Increased afterload is evidenced by the presence of increased serum B-natriuretic peptide in infants born growth restricted (67).

Where placental insufficiency is present, the fetal heart must also adapt to a reduced supply of glucose, with the fetal heart producing ATP from glycolysis and oxidation of lactate. Despite this, cardiac glucose consumption is not altered in growth restriction due to increase in insulin receptor GLUT4 in the heart, which increases insulin transport to maintain glucose consumption (68). Thus, glucose availability is not considered a primary limiting factor for fetal cardiac function. More in-depth analysis of the effects of suboptimal oxygen and glucose supply to the developing heart can be examined in animal studies of FGR (69). These studies show that the fetal heart is remodeled in a manner similar to that seen in dilated cardiomyopathy. Cardiomyocyte development is adversely affected and programmed cell death is increased in growth restricted fetal guinea pigs and sheep, with a persistence of the mononucleated, primitive cell type (70, 71). Permanent alterations in heart morphology are detected into adulthood, as evidenced by persistence in the deficits in cardiomyocyte number and cardiac hypertrophy (70).

The transition to ex utero represents a particularly critical period where the heart must rapidly adapt to new pressure and flow demands. After birth, the external pressures around the heart are reduced, due to alleviation of the liquid-filled lungs and amniotic fluid. Concurrently, the low resistance placental circulation is removed, temporarily decreasing cardiac output and increasing afterload, and thus heart rate, end-diastolic pressure and stroke volume must all be increased to maintain adequate cardiac output. In response to these altered pressure demands throughout the transition to ex-utero life, the myocardium undergoes rapid changes in cardiac muscle protein expression (72). One critical change precipitated by such pressure changes at birth is a shift in the fibrous component of sarcomeres toward smaller isoforms, which increase the passive tension within with postnatal heart (72). It is postulated that the growth-restricted fetus undergoes these changes *in utero*, due to the presence of increased afterload secondary to high placental resistance, and resulting in altered cardiac compliance (73). In human infants and in experimental animal models of FGR, the heart is shown to have shorter sarcomeres, which likely contributes to decreased contractile strength (73, 74). Further, changes in the large sarcomere protein titin are described in the FGR heart, reflecting a shift from a large compliant isoform toward a small and stiff isoform (73). As titin is a major determinant of sarcomere length, this change in isoform is consistent with overall reduction in sarcomere length in the hearts of growth-restricted fetuses, and has consequences for cardiac development and function.

Changes in the hearts of growth-restricted fetuses are directly coupled with changes in the wider cardiovascular system, notably the vasculature. It is now well described that vascular responses to placental insufficiency and chronic hypoxia are vascular bed-dependent. In peripheral vascular beds, human and animal data show that sustained vasoconstriction and peripheral vascular resistance in response to chronic hypoxia induces arterial stiffness and elevated central pulse pressure (75–77). Growth restriction induced via chronic hypoxia increases peripheral vascular tone via numerous methods, including endothelial dysfunction (76), increased sympathetic nervous system activation (69) and oxidative stress (78). Oxidative stress, induced via increased reactive oxygen species generation, quenches nitric oxide (NO), thereby reducing its bioavailability and increasing peripheral vascular tone. We have previously described an increase in plasma urate levels arising from chronic fetal hypoxia (78) suggesting activation of a potent oxidative enzyme, xanthine oxidase. Importantly, it is this altered vascular tone in fetal life that sets up developmental programming for future hypertension, as evidenced in both FGR animals (79) and human cohorts (61). Central vessels, such as the aorta and carotid arteries, have increased wall thickness (80) and increased stiffness (81) in FGR humans and animals. The vascular changes described above persist into adulthood, however, they are more pronounced in peripheral vascular beds compared to central vascular bed (82).

Vascular compensation is observed in FGR offspring, wherein remodeling of the arterial wall, collagen and elastin content contribute to altered vascular mechanics (83). Rodent and guinea pig studies show that interruption of fetal growth in mid gestation coincides with a crucial period of elastin production within vasculature, attenuating elastin deposition and subsequently content, such that elastin is reduced and collagen increased (84, 85). This remodeling greatly impacts on vascular mechanics, as collagen is 100-times stiffer than elastin and, as a consequence, vascular stiffness is significantly increased (85). These changes in vessel biomechanics are most notably in the lower body arteries of growth-restricted offspring (83). Following low protein diet restriction, the aorta from adolescent rodents are not only stiffer, they also have increased fibrotic tendency, despite being normotensive (67). However, a more profound effect on vascular extracellular matrix remodeling is seen with placental dysfunction-induced growth restriction, compared to other factors such as diet (high fat) or fetal sex. These data are suggestive that vascular remodeling occurs primarily in response to changes in pressure and flow caused by chronic hypoxia and adaptive hemodynamic redistribution, rather than metabolic or hormone alterations.

#### Impact

The evidence presented above all indicates that exposure to placental insufficiency and chronic hypoxia significantly alters *fetal* development of the cardiovascular system. Unsurprisingly, the fetal cardiovascular alterations subsequent to placental insufficiency persist into clearly detectable structural and functional changes in the early postnatal period. After birth, tissue Doppler imaging (TDI) has allowed detection of persistent sub-clinical changes in movement and timing of the myocardium throughout the cardiac cycle, in particular during myocardial relaxation (86). In the first days of life, infants who were growth restricted show altered cardiac structure detectable on ultrasound with decreased sphericity index (a more globular shape), together with increased interventricular septum and left ventricle wall thickness (77). Further, load-dependent diastolic function is impaired (77, 87) this often represents impaired cardiac relaxation resulting in the transition from contraction to relaxation occurring prior to aortic valve opening, a situation which is common in hearts exposed to chronically high afterload. Frequently, FGR does not alter overall cardiac output, however components of cardiac output are altered with decreased stroke volume and increased heart rate often presenting in the FGR newborn (86). With increasing severity of FGR there is increased biomarkers of myocardiac cell damage, such as heart fatty acid binding protein (H-FABP), and incremental worsening of both systolic and diastolic dysfunction and in particular heart relaxation is altered (66). These alterations are indicative of hemodynamic compromise and are linked to worsening outcomes including fetal demise (88). Early signs of alteration in blood pressure in association with FGR remains contentious—we have documented increased blood pressure in the early postnatal period (80), whilst others show no change in blood pressure (82); these differences may reflect the difference between clinical and pre-clinical studies or the severity of the growth restriction induced.

In turn, *in utero* cardiac and vascular remodeling in FGR neonates programs for cardiovascular disease into adulthood. Indeed, the consequences of growth restriction on adult cardiovascular function are now well studied, and are central to the Developmental Origins of Health and Disease (DOHAD) hypothesis. These findings are apparent from both human epidemiological and experimental paradigms in growth-restricted offspring (76, 85) and adults (60). Long-term evidence of the link between low birth weight and developmental programming is available in the infants born in famine conditions in Europe in the 1900s wherein SGA is linked with significantly higher blood pressure in later life (89), and with increased risk of ischemic heart disease and cerebrovascular disease (90). Precursors of long-term suboptimal outcomes such as stroke and hypertension (91) have been proposed to be evident in growth restriction offspring as pre-atherosclerotic vascular damage in both newborns (92) and 18 month old FGR offspring (93).

Despite excellent evidence of the link between FGR and adult cardiovascular disease, there is some difficulty in dissociating the potentially separate effects of placental insufficiency/FGR and preterm birth. Growth restricted fetuses are often born preterm, particularly early-onset severe FGR infants, and preterm birth is also associated with adverse effects on the developing cardiovascular system (94). A recent study by Cohen et al. (87) followed both preterm and preterm FGR infants to 6 months of age to determine cardiac morphology. They found that changes in cardiac structure and function associated with preterm birth alone were sub-clinical, and normalized in childhood, while only thickened ventricular walls persisted into 6 months of age in FGR infants (87). This study goes some way to delineate the separate effects of prematurity and growth restriction and suggests a possible persistence of structural changes in FGR over and above the effects of prematurity.

### Respiratory Morbidity

#### Clinical Features

There is heterogeneity in descriptions of pulmonary complications associated with FGR, which probably reflect the heterogeneity in growth restriction itself. There is however good evidence that chronic hypoxia associated with FGR interrupts normal pulmonary development, and increases susceptibility to both short- and long-term respiratory compromise. Preterm FGR newborns are 45% more likely to have bronchopulmonary dysplasia (BPD) or die from respiratory complications after birth as compared to well-grown infants (45). Further, even FGR infants born at term have worse respiratory outcomes than appropriately grown infants (95). FGR infants spend a significantly increased time in NICU and on mechanical ventilation compared to age-matched control infants, and rates of respiratory distress syndrome (70) and BPD are increased with FGR (45, 96, 97). Indeed, large multicenter trials for early-onset FGR describe that BPD is the most common morbidity for this population. The risk of BPD is greater when FGR and preterm birth are co-morbidities. Growth restriction is also associated with pulmonary hypertension of the newborn (96). FGR is associated with impaired lung function in children (98) that can persist to adulthood (99).

#### Pathophysiology

Human FGR cohort studies and preclinical animal studies describe that FGR can result in altered lung development; in some cases these are subtle structural and/or biochemical changes, wherein the timing and severity of compromise modulates effect. In animal studies, an early onset placental or hypoxic compromise mediates a more pronounced adverse outcome. Chronic hypoxia in fetal sheep resulting in FGR induces an adaptive response within the developing lung, where genes regulating hypoxic signaling, lung liquid reabsorption and surfactant maturation are increased (100). A 2-week exposure to hypoxia alone in rats disrupts alveolarization, reducing alveolar number via reduced septation (101). In fetal sheep we have induced late-onset placental insufficiency and FGR to examine lung morphology in preterm and term-born lambs. Lambs born naturally at term have simplified lung architecture with decreased secondary crest abundance and increased elastin deposition (102). Lambs that are delivered preterm and exposed to 2 h of mechanical ventilation do not demonstrate a difference in lung structure between FGR and appropriately-grown lambs, with no difference in the ratio of lung tissue to airspace or septal crest density, however the early tissue injury marker cyr61 is significantly increased in FGR lambs (103). Further, we observed that both FGR and appropriately grown lambs had similar ventilation requirements in the first hours of life. These findings extend previous results in FGR animal experiments from our group (104) and others (105, 106), which find no overt difference in pulmonary structure of FGR offspring. When we compare results between our preterm and term lamb cohorts, it is evident that the timing and duration of placental insufficiency is a critical determinant of lung dysfunction. We have recently examined the effects of early-onset placental insufficiency on lung structure and function, finding that lung cellular morphological changes are present (unpublished results). Accordingly, we propose that altered lung structural development is dependent on the timing of compromise, rather than the severity of growth restriction. Further, in early-onset FGR, the severity of fetal hypoxia has an inverse relationship with pulmonary surfactant production leading to decreased surfactant, a relationship not maintained in late-onset FGR (107). It is well accepted that without adequate surfactant, the newborn is at increased risk of pulmonary complications after birth, particularly when the infant is born preterm.

As discussed above, chronic hypoxia results in the fetal adaptive response of redistribution of cardiac output. MRI studies have confirmed that in late gestation of human fetuses, growth restriction is associated with an increased superior vena cava flow and, consequently, decreased pulmonary artery flow (108). This hemodynamic response is contributed by increased pulmonary vascular resistance (97). Increased pulmonary vascular resistance also reduces venous return to the left heart (109) and enhances right ventricular afterload. Combined ventricular output is thus maintained (108). Postnatally, FGR does not alter pulmonary blood flow during the transition to ex utero life, but left ventricular output is lower (110). Thus it is apparent that the hemodynamic adaptation to chronic hypoxia also has important implications for pulmonary vascular development, and accordingly, lung structure and function in FGR offspring.

A handful of studies have also examined fetal breathing movements in the developing fetus, and undertaken comparison in FGR versus appropriately grown fetuses. Fetal breathing movements are an important component of normal lung development, as they provide a stretch stimulus for growth throughout gestation (111). In FGR sheep, fetal breathing movements are significantly reduced in late gestation, although it is noted that not all experimental models of placental insufficiency show such changes (69). The cessation of fetal breathing movements in response to placental insufficiency is thought to occur by way of physiological response to reduce metabolic rate and thus conservation of oxygen, and is associated with disrupted alveolarization (112).

Deficits in pulmonary development subsequent to placental compromise are not confined to lung alveolar morphology. There is a growing understanding of the link between poor alveolar development and poor lung vascular development (113–115), called the *Vascular Hypothesis*. Growth restriction, induced via hyperthermia in pregnant sheep, impairs both lung alveolar and vascular development in the developing fetus (116). In complimentary experiments, lung alveolar cells isolated from the same growth-restricted fetuses demonstrate reduced cell growth, migration and branching, which are key components of normal lung development (116). These findings are confirmed *in vivo* in which growth-restricted offspring demonstrate diminished pulmonary vascular function and density, together with decreased pulmonary alveolarization (116, 117). Abnormal pulmonary vascular development in growth restricted fetuses is likely to be a key mechanism increasing the risk of BPD, pulmonary hypertension and life-long reduction in respiratory capacity, such as seen in chronic obstructive disease (116).

#### Impact

BPD is a chronic lung disease characterized by arrested airway and parenchymal development and resulting in long-term respiratory complications, with a high susceptibility in preterm and growth restricted infants. BPD is a multifactorial condition, however it is primarily thought to result from chronic ventilation-induced injury in preterm infants, contributed by lung exposure to excess oxygen and inflammation. FGR is an independent risk factor for BPD in human infants (118–120). Being born subsequent to placental insufficiency and growth restricted is associated with a 3.6-fold higher risk of developing BPD than age-matched control infants (120), despite FGR infants having similar RDS rates as appropriately-grown counterparts. Lio et al. (121) have also recently shown that FGR infants with placental dysfunction have a 6-fold increased risk of developing BPD compared to low birth weight/ SGA infants. Further, they noted that birth weight *per se* and not ventilation duration, or other neonatal morbidities, contributed to the presence of BPD. Maternal vascular unit deficiency, a marker for pre-eclampsia, is a common placental pathology associated with FGR, and it has also been shown that maternal vascular unit dysfunction doubles the risk of BPD in preterm human infants (118). Thus, human and animal data strongly support that the foundations of postnatal lung deficits and BPD are laid down *in utero* in FGR infants with placental insufficiency, and that vascular pathology is likely to be a contributing factor.

Neonatal pulmonary hypertension is highly associated with decreasing gestational age and low birth weight, and is a common complication of BPD (96). Pulmonary hypertension is characterized by hypoxemia of the newborn and right-to left shunting through the ductus arteriosus, due to maintenance of high pressures within the pulmonary circulation. Accordingly, neonatal pulmonary hypertension occurs via a failure of structural cardiovascular remodeling after birth, and is likely developmentally programmed *in utero* (122). In post mortem tissue analysis it is shown that newborns with pulmonary hypertension displayed reduced pulmonary vascular surface area with increased muscularization of distal pulmonary vasculature (123). These data suggest a strong association with FGR induced by vascular remodeling in chronically hypoxic fetuses, resulting in impaired control of vascular tone within the pulmonary circulation after birth. Altered pulmonary vascular composition has been more closely examined in growth restricted rats, demonstrating increased pulmonary vasoconstriction caused by local endothelial dysfunction and excessive collagen and reduced elastin in the pulmonary vasculature (124). Animal models of FGR also provide strong evidence that the hallmarks of pulmonary hypertension are already present in the growth-restricted fetus and offspring soon after birth. Our group and others have shown vascular changes, including decreased vascular density and dysfunction in fetal sheep (116) and in 2-h-old lambs (110). Thus, even prior to birth, FGR is associated with pulmonary hypertension.

The long-term effects of low birth weight have been examined in adult offspring conceived during the 1940s Dutch famine, who show an increased risk of obstructive airway disease (89). Further analysis of this cohort determined that neither serum immunoglobulin E concentration nor mean lung volumes were different (125). The authors speculate that bronchial reactivity must be the cause of the airway disease following growth restriction.

### Neurological Morbidity

#### Clinical Features

FGR is strongly linked to suboptimal brain development, and long-term neurological dysfunctions in motor ability, cognition and learning, and behavior. We have recently reviewed the consequences of placental insufficiency and FGR on the developing brain (28), and describe that the age of onset and severity of FGR, together with gestational age at birth, play important modulatory roles in altered brain structure and function. The first indication of structural anomalies of the FGR brain can be derived from magnetic resonance imaging (MRI) during fetal development. MRI of the fetal brain during development demonstrates reduced brain volume, and altered cortical folding and brain morphology in FGR fetuses (126, 127). Arthurs et al. (128) showed lower diffusion weighted imaging values in parts of the brain in severe FGR fetuses as compared to normal age-matched controls, which were suggestive of an abnormal maturational profile. Postnatally, at term-equivalent age, MRI detects reduced intracranial volume, particularly contributed by decreased cortical gray matter volume in FGR infants (129), and altered developmental profile of white matter myelination (130), the hippocampus (131) and the basal ganglia (132) of growth-restricted infants, compared to appropriately grown infants. Functional MRI is also an upcoming tool to study whole brain functional networks in newborn infants for the assessment of altered organization and prediction of long-term neurodevelopment (133). Diffusion tensor imaging (DTI) and connectivity-based analysis of the FGR brain in the neonatal period is also being increasingly investigated (134).

MRI has the ability to detect even relatively small volume, structural, and organizational differences within the brain of FGR and appropriately-grown infants (135) but MRI capability and expertise in analysis is not readily available at all birth centers. In contrast, neonatal cranial US is widely used, but shows less sensitivity for detection of these subtle, but important neurological changes associated with neuropathology in FGR infants (136). Cranial ultrasound is frequently used as an assessment tool in premature infants, and term infants with severe FGR, to identify significant neuropathology in the neonatal period. There remains uncertainty as to whether cranial ultrasound can adequately detect neuropathology associated with FGR when compared to age-matched appropriately grown preterm infants (135, 136). Certainly in older preterm and term FGR infants, the benefit of routine cranial ultrasound screening in the neonatal period is questionable (137). We did not find evidence of altered cerebral ventricular volume using ultrasound imaging in FGR infants <10th centile, however we did observe a correlation between increasing ventricular volume and a decrease in functional motor scores (138). Cruz-Martinez et al. (139) have suggested that FGR infants with signs of middle cerebral artery and other Doppler abnormalities (indicative of significant brain sparing) are more likely to have neuropathology that can be detected on neonatal cranial ultrasound. This is interesting, as it further supports that the term *brain sparing* is a misnomer, and while it represents an appropriate survival response in the fetus, it is actually associated with worsening fetal condition and greater brain injury (28).

FGR infants frequently have a reduced head circumference compared to age-matched appropriately-grown infants, which is likely due to reduced brain volume (129), and reduced brain volume persists to 12 months of age (140). Cerebellar and hippocampal volumes may also be reduced (130). Brain myelination and connectivity have been shown to be adversely affected in FGR infants in the first 12 months of life, representative of white matter injury (141). Diffusion MRI of the human brain shows that the overall neuronal network complexity and connectivity of the FGR brain is reduced, with reduced global and local axonal circuits (142). Long-range cortical-basal ganglia (thalamocortical) connections are decreased in children born preterm with FGR, compared to children born preterm but appropriately-grown (142), indicating that brain connectivity is significantly worse in children who were FGR compared to children who were preterm but well-grown. Deficits in brain connectivity correlate with neurobehavioral impairments including hyperactivity and poor cognition at school in children who were born FGR (143).

Neonatal functional assessment may detect early problems with neurological processing and behavior in infants who were born growth restricted. Tolsa et al. (129) showed that FGR newborns had specific alterations of brain structure as studied by volumetric MRI at preterm and term age, with reduced cortical gray matter volume correlating with deficits in attention and responsivity at term-equivalent age. General movement assessments (Prechtl movements) provide an early motor analysis, wherein abnormalities are predictive for cerebral palsy, and general movements may be adversely affected in some FGR infants (144). Similarly, electroencephalography performed early in the neonatal period has been shown to be affected and may correlate with adverse neurodevelopment in studies of FGR infants (145, 146). There is however limited data on early detection of functional deficits in growth-restricted infants, reflecting challenges in detecting delayed neurodevelopment in the neonatal period.

#### Pathophysiology

It is now well established that the traditional brain sparing physiology does not necessarily mean normal cerebral development *in utero* (28). In fact, fetuses with the most severe brain sparing are at the highest risk of adverse neurodevelopment in childhood. Prenatal loss of vasoreactivity in FGR has been suggested as a mechanism for poor outcomes, in which fetuses who do not adjust their cerebral circulatory control in response to hypoxic challenge may be more at risk of impaired cerebrovascular regulation (147). There are also reports of preferential perfusion and cerebral redistribution of brain blood flow in FGR fetuses, leading to some brain regions being at higher risk of injury (148). This is supported by work in fetal sheep to demonstrate that FGR is associated with regional cerebral blood flow redistribution, with the most notable differences between FGR and appropriately grown fetuses seen in the cerebral cortex and periventricular white matter (21).

Cerebral blood flow frequently continues to be abnormal for the first few days after birth in FGR human infants, but whether this puts infants at an increased risk of acute brain injury is not known. It has been reported the cerebral blood flow remains elevated after birth in FGR infants (149), even when the neonate is no longer exposed to a hypoxic environment and is no longer in need of a compensatory change in cardiac output. Postnatally, elevated cerebral blood flow might potentiate hyperoxia and oxidative stress within the fragile brain, which could also contribute to further neurological damage.

Animal models of chronic hypoxia and growth restriction have helped us to understand the development of neuropathology associated with placental insufficiency and FGR (28, 150–152). Adverse effects on brain gray matter development, white matter, and cerebellum have been described both in sheep, rabbit and rat models of FGR (153–155). In fetal sheep, we showed that early-onset placental dysfunction is associated with more widespread and severe white matter brain injury and neuroinflammation compared with late-onset, however both early- and late-onset FGR demonstrate complex patterns of gray and white matter neuropathology (154). Animal studies also show that the severity of brain injury, and the resultant neurodevelopment deficits, depends on the extent and severity of brain involvement in FGR (156). Hypomyelination and delayed myelination due to oligodendrocyte maturational deficits have been identified as possible mechanisms causing the white matter injury seen in FGR infants (157). Deficits in neuronal connectivity have also been described in animal models (158). Our group, and others, has observed that deficits in various components of the neurovascular unit play a significant role in the brain injury seen in animal models of FGR (159). Prematurity is a confounder in human FGR, but studies in FGR animals allow the separation of growth restriction and preterm birth. The individual contributions of preterm birth and/or neonatal ventilation of the FGR newborn on the progression of brain injury are now being examined (160, 161). These studies have determined that preterm birth and ventilation synergistically predispose the vulnerable FGR brain to neuropathology.

#### Impact

FGR infants are at increased risk of adverse neurodevelopmental outcomes in childhood. Neurological morbidities related to motor deficits, including cerebral palsy, behavioral issues, and cognitive impairment is significantly increased in young children and adolescents who were diagnosed as growth restricted at birth (28, 162–164). The risk of cerebral palsy is 30-fold greater in FGR infants, compared to those that are well grown (165), and increases with worsening growth restriction. Overall, >40% of children who have cerebral palsy had a low birth weight; that is, they were growth restricted, born preterm, or both (166). This is important, as FGR and preterm birth are frequent co-morbidities. In addition to motor deficits, preterm FGR infants followed-up at 1, 2, and 3 years of age showed deficits in cognition and behavioral outcomes compared to preterm age-matched appropriately-grown infants (167). Further, a longitudinal study observing FGR offspring with evidence of brain sparing from birth to middle school age (9–10 years old) found a complex set of neurodevelopmental deficits, such as a significant reduction in IQ, compared to age-matched appropriately-grown children (168). Multiple follow-up studies of FGR infants into school age describe diminished gross and fine motor skills, cognition, memory, and academic ability, as well as neuropsychological dysfunctions encompassing poor attention, hyperactivity and altered mood (143, 169–171). FGR infants born preterm and those with fetal circulatory redistribution are at the greatest risk for the worst outcomes (172). These adverse outcomes can continue into adolescence and young adulthood (173). It is apparent that determining the neurodevelopmental consequences of FGR is complicated by the severity of FGR, early- or late-onset, and the gestational age at delivery (28). However, in both early- and late-onset FGR, the presence of cardiovascular redistribution and brain-sparing is associated with abnormal neurodevelopmental outcomes (28).

### Interventions for Improved Outcomes

Management of pregnancies complicated by FGR represents a balance between antenatal compromise, often with worsening chronic hypoxia that contributes to subpotimal organ development, and the risks associated with preterm delivery and postnatal intensive care, which may also contribute to morbidities. In high-income countries, about half of fetuses with moderate- to severe-growth restriction are detected antenatally and are therefore amenable to treatment during pregnancy, but it remains that nearly 40% of infants born at the 3^rd^ centile for weight are not detected *in utero* (44). With this in mind, both antenatal and postnatal therapies must be considered. Currently, no specific treatment is available for FGR. Potential treatments should target maldevelopment of multiple organs, various injurious pathways, cell types, and structural deficits that manifest over different developmental stages. Here we will provide an overview of the current state of understanding for a handful of treatments for FGR (Figure 3).

#### Antenatal

Antenatal treatments are principally aimed at improving placental function and thereby increasing fetal growth *in utero*. To date, the best studied of these has been sildenafil citrate. Sildenafil is a potent phosphodiesterase type 5 (PDE5) inhibitor that is an effective smooth muscle relaxant where the PDE5 enzyme is present in an organ or tissue, as is the case for the human placenta (174). The effects of sildenafil on smooth muscle are mediated via an enhanced and prolonged nitric oxide release leading to vasodilatation. Both *in vitro* and *in vivo* studies demonstrate that sildenafil vasodilates human myometrium vessels from normal (175, 176) and growth restricted placenta. Most experimental studies to date support that sildenafil increases fetal weight in compromised rat, sheep and human pregnancies (177). In contrast, we have shown that antenatal sildenafil administration to pregnant sheep with placental insufficiency decreases fetal weight and worsens fetal hypoxia (178). Although initial preclinical evidence for the multinational STRIDER trial suggested improved outcomes for FGR infants, this trial has now been aborted due to unexpected baby deaths (179), leading to a call for increased preclinical studies underpinning clinical trials (180), and improved understanding of the effects of sildenafil on the fetus given that it crosses the placenta (181). The longer acting tadalafil remains an active clinical experimental treatment of interest as an antenatal therapy for FGR and, given that tadalafil does not cross placenta (174), it may be more favorable as a targeted placental treatment.

The EVERREST Project is also investigating a targeted approach to improve placental function in pregnancies complicated by FGR using gene therapy to inject vascular endothelial growth factor (VEGF) into uterine arteries (182). VEGF is known for its role in inducing angiogenesis and in the EVERREST Project it is hypothesized that application of adenovirus VEGF in, or near placental arteries will induce a local and acute increase in VEGF expression, and subsequent angiogenesis of the placental vasculature. Preclinical studies have shown promise with improved blood flow (183) and fetal weight gain (184) in animal models of growth restriction, resulting from the improved vascularization of the placenta. The clinical trial is ongoing.

A recent large animal (sheep) study examined intra-amniotic administration of the growth-promoting protein insulin-like growth factor-1 (IGF-1) (16). This work showed that increasing the bioavailability of IGF-1 in pregnancies complicated by placental insufficiency and FGR improved birth weight in female lambs, but not males, and modified postnatal catch-up growth in both females and males. Intrauterine IGF-1 also mediated expression of key somatotrophic and metabolic genes, indicative that antenatal treatment could be utilized to positively affect postnatal growth and wellbeing.

A number of antenatal treatments have been explored preclinically that aim to restore fetal oxidative tone via maternal antioxidant administration, using agents such as allopurinol, melatonin and vitamin C (75, 79, 185). Antioxidant treatment has principally targeted improved cardiovascular and neurological outcomes in growth-restricted offspring. To date, melatonin has been the most widely studied, given melatonin's established safety profile, ease of administration, and strong antioxidant benefits. In sheep, we have shown that maternal melatonin administration to ewes carrying a growth restricted fetus results in a significant improvement in vascular function and reduced arterial stiffness, two vital pathologies evident in FGR offspring, which predispose to cardiovascular disease (75). Melatonin administration also resulted in improved cardiac function in the right ventricle. Further, this study showed that maternal melatonin improved fetal oxygenation and increased birth weight (75), however other ovine studies show either no improvement in birth weight (186) or exacerbation of growth restriction with melatonin (187). In cultured human umbilical vein endothelial cells (HUVECs), melatonin improves vascular endothelial integrity, likely via combined anti-oxidant and anti-inflammatory mechanisms (188). Exposure to antenatal melatonin does not reverse alveolar simplification in FGR newborn lambs (102), but does improve pulmonary vascular structure and function (189), and pulmonary tone may be maintained long term via alteration to receptor populations (190). As our understanding of perturbations to lung growth in FGR offspring continues to be explored, so too does the opportunity for targeting novel pathways. For example, recent work has shown that NPY is down regulated in FGR, where NPY is a sympathetic neurotransmitter that is critical for normal lung growth (191).

The effects of maternal melatonin administration on brain development have also been examined. Antenatal melatonin crosses the placental and the blood brain barrier, and melatonin is a strong antioxidant and also demonstrates anti-inflammatory benefits in the developing brain (186, 192–194). In pregnancies complicated by placental insufficiency and FGR, maternal melatonin improves white matter brain development via increased myelination and decreased axonopathy in the fetal brain, and subsequently, neurobehavior of FGR+MLT lambs is significantly improved after birth (186). Melatonin has also been shown to have beneficial effects on cerebral vasculature by preventing FGR-related apoptosis and disruption of blood brain barrier instability via improved vascular interactions with astrocytes and pericytes (195). Antenatal melatonin has been examined in pilot studies to treat FGR (186) and preeclampsia (188) with results supporting that melatonin is an effective anti-oxidant that is safe for the mother and baby, and may extend pregnancy (196).

Emerging evidence supports that the glycoprotein lactoferrin shows potential as an antenatal treatment for pregnancies complicated by FGR, particularly for the developing brain (197). Lactoferrin is a glycoprotein that demonstrates strong antioxidant, anti-inflammatory and anti-microbial effects—important factors that could mediate neuroprotective benefits. In rats, lactoferrin supplementation during pregnancy shows positive benefits for dexamethasone-induced fetal growth restriction (197). Maternal lactoferrin significantly increased birth weight of control rat pups, and FGR offspring exposed to lactoferrin showed a normalized weight at postnatal day 21. Lactoferrin supplementation also improved brain hippocampal structure and stimulated brain derived neurotrophic factor (BDNF) (197), important observations in light of the neuropathology associated with human FGR. Nutritional supplementation (glucose, amino acids and electrolytes) into the amniotic sac of FGR rabbits has also recently been explored, with some promising results suggesting that survival rate for FGR offspring was improved with treatment, although birth weight and cardiac function deficits were not improved (198).

#### Postnatal

As mentioned above, nearly 40% of human infants with severe FGR are not detected antenatally (44), and therefore not amenable to antenatal treatments. In light of this we must continue to investigate therapies to improve multi-organ dysfunctions in growth restricted infants. We have highlighted in this paper that deficits in cardiovascular, pulmonary and cerebral development are already present at birth in FGR infants, principally caused by chronic hypoxia *in utero*. Therefore any potential postnatal therapy would aim to be reparative and to prevent progression of ongoing multi-organ damage.

Lactoferrin shows great potential as a postnatal therapy, in addition to positive effects antenatally. Lactoferrin is highly abundant in human colostrum and milk, and it reaches the brain after oral administration (197, 199). In this regard, breastfed infants show higher total anti-oxidant capacity and a lower oxidative stress index compared to non-breastfed infants. Importantly, randomized controlled trials with nutritional lactoferrin supplementation in premature neonates demonstrate a promising reduction in late onset sepsis and necrotizing enterocolitis (200). Lactoferrin supplementation during lactation is protective for neonatal rats exposed to either hypoxia-ischemia or lipopolysaccharide-induced systemic inflammation (201, 202). Analysis of the neonatal rat brain using a combination of advanced MRI analysis and histology demonstrates that gray and white matter microstructure is normalized with lactoferrin supplementation, myelination is protected and measures of axonal integrity and brain organization are restored in rats with lactoferrin supplementation (201). Early environment enrichment or postnatal stimulation has also been shown to have some benefits in brain connectivity in a rabbit model of FGR (203). While there are no current published studies on stem cell therapy for FGR related brain injury, our lab and others are working on testing their applications in FGR and other pregnancy complications (204). The application of postnatal therapies to improve multi-organ deficits associated with FGR should remain a foremost preclinical research area.

## Conclusions

Understanding the pathophysiological mechanisms that underlie neonatal morbidities that are particularly associated with FGR provide the fundamental basis for improving short- and long-term outcomes in growth restricted offspring. It is clear that placental compromise and chronic fetal hypoxia program the fetus for suboptimal growth and development, with fetal cardiovascular dysfunctions and altered organ development already apparent in the FGR fetus during pregnancy. The timing of the onset of placental insufficiency, the severity of growth restriction, the degree of cardiovascular adaptation, and gestational age at birth are all critical factors that modify outcome for FGR infants. In the neonatal period, FGR infants demonstrate early evidence of cardiac, vascular, pulmonary, neurological and other deficits, which can lead to long durations in neonatal intensive care, and long-term health problems. Improved antenatal detection, and both antenatal and postnatal therapies that target the key pathophysiological mechanisms underlying altered multi organ structure and function must be considered critical research areas.

## Author Contributions

AM and BA critically researched literature, co-wrote the first draft of the manuscript and approved the final version. MC-M, GJ, GP, and SM reviewed the manuscript, contributed to various sections and approved the final version.

### Conflict of Interest Statement

The authors declare that the research was conducted in the absence of any commercial or financial relationships that could be construed as a potential conflict of interest.
